# Pulmonary vein isolation using pulsed field ablation vs. high-power short-duration radiofrequency ablation in paroxysmal atrial fibrillation: efficacy, safety, and long-term follow-up (PRIORI study)

**DOI:** 10.1093/europace/euae194

**Published:** 2024-07-12

**Authors:** Nico Reinsch, Anna Füting, Stefan Hartl, Dennis Höwel, Eva Rausch, Yali Lin, Karampet Kasparian, Kars Neven

**Affiliations:** Department of Electrophysiology, Alfried Krupp Krankenhaus, Alfried-Krupp-Straße 21, 45131 Essen, Germany; Department of Medicine, Witten/Herdecke University, Alfred-Herrhausen-Straße 50, 58448 Witten, Germany; Department of Electrophysiology, Alfried Krupp Krankenhaus, Alfried-Krupp-Straße 21, 45131 Essen, Germany; Department of Medicine, Witten/Herdecke University, Alfred-Herrhausen-Straße 50, 58448 Witten, Germany; Department of Electrophysiology, Alfried Krupp Krankenhaus, Alfried-Krupp-Straße 21, 45131 Essen, Germany; Department of Medicine, Witten/Herdecke University, Alfred-Herrhausen-Straße 50, 58448 Witten, Germany; Department of Medicine, Witten/Herdecke University, Alfred-Herrhausen-Straße 50, 58448 Witten, Germany; Department of Cardiology, St. Marienhospital Vechta, Vechta, Germany; Department of Electrophysiology, Alfried Krupp Krankenhaus, Alfried-Krupp-Straße 21, 45131 Essen, Germany; Department of Medicine, Witten/Herdecke University, Alfred-Herrhausen-Straße 50, 58448 Witten, Germany; Department of Electrophysiology, Alfried Krupp Krankenhaus, Alfried-Krupp-Straße 21, 45131 Essen, Germany; Department of Medicine, Witten/Herdecke University, Alfred-Herrhausen-Straße 50, 58448 Witten, Germany; Department of Medicine, Witten/Herdecke University, Alfred-Herrhausen-Straße 50, 58448 Witten, Germany; Department of Oncology, Gastroenterology and Hematology, Alfried Krupp Krankenhaus, Essen, Germany; Department of Electrophysiology, Alfried Krupp Krankenhaus, Alfried-Krupp-Straße 21, 45131 Essen, Germany; Department of Medicine, Witten/Herdecke University, Alfred-Herrhausen-Straße 50, 58448 Witten, Germany

**Keywords:** Pulsed field ablation, Pulmonary vein isolation, Atrial fibrillation, High-power short-duration ablation, Ablation index

## Abstract

**Aims:**

Pulsed field ablation (PFA) is a novel, non-thermal, cardiac tissue-selective ablation modality. To date, radiofrequency (RF)-guided high-power short-duration (HPSD) ablation represents the gold standard besides cryo-ablation for pulmonary vein isolation (PVI). This single-centre, retrospective study investigated the efficacy of PFA-PVI vs. HPSD-RF PVI in terms of single-procedure arrhythmia-free outcome and safety in a real-world setting.

**Methods and results:**

Consecutive, paroxysmal atrial fibrillation (AF) patients who underwent PVI using PFA or HPSD-RF were enrolled. In group PFA, PVI was performed using a pentaspline PFA catheter. The ablation procedure in group HPSD-RF was performed with RF energy (45 W, ablation index). A total of 410 patients (group PFA, 201; group HPSD-RF, 209) were included. There was no difference between both groups regarding age, gender, and CHA_2_DS_2_-VASc score. The procedure time was significantly shorter in group PFA [61 (44–103) vs. 125 (105–143) min; *P* < 0.001]; fluoroscopy time and dose area product were significantly higher in group PFA [16 (13–20) vs. 4 (2–5) min; *P* < 0.01 and 412 (270–739) vs. 129 (58–265) μGym^2^; *P* < 0.01]. The overall complication rates were 2.9% in group PFA and 6.2% in group HPSD (*P* = 0.158). There was one fatal stroke in the PFA group. The 1-year Kaplan–Meier estimated freedom from any atrial tachyarrhythmia was 85% with PFA and 79% with HPSD-RF (log-rank *P* = 0.160). In 56 repeat ablation procedures, the PV reconnection rate was 30% after PFA and 38% after HPSD-RF (*P* = 0.372).

**Conclusion:**

Both PFA and HPSD-RF were highly efficient and effective in achieving PVI in paroxysmal AF patients. The arrhythmia-free survival is comparable. The PV reconnection rate was not different.

## Graphical Abstract

**Graphical Abstract:**
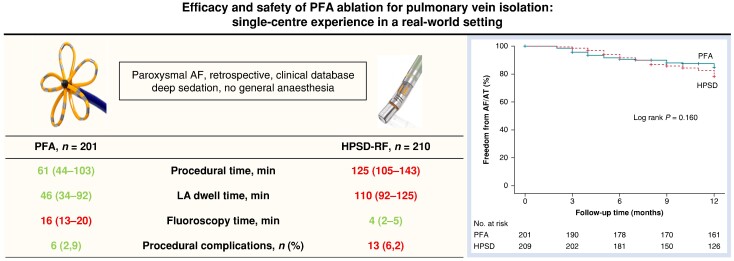

**What’s new?**

- Real-world performance of the pentaspline pulsed field ablation (PFA) catheter in a paroxysmal, all-comer patient population.
- Largest, single-centre comparative analysis of PFA vs. high-power short-duration (HPSD) radiofrequency (RF) ablation.
- Similar, high acute and long-term efficacy of PFA and HPSD-RF ablation.
- Pulsed field ablation yielded significantly shorter procedural and left atrial dwelling times compared to RF ablation. However, 3D electroanatomical mapping to guide RF-based procedures contributed to significantly shorter exposure to fluoroscopy.
- Both ablation strategies showed a low risk of serious complications.
- At the time of repeat ablation, PFA patients showed a non-significantly lower number of reconnected pulmonary veins compared to those initially treated with HPSD-RF.

## Introduction

Atrial fibrillation (AF) is the most common arrhythmia causing significant morbidity and mortality.^1^ Catheter-based ablation of the pulmonary veins (PVs) is an established treatment for rhythm control in paroxysmal AF.^2^ Point-by-point radiofrequency (RF) ablation and cryo-balloon ablation are the most common techniques to achieve pulmonary vein isolation (PVI). In case of RF-guided ablation, high-power short-duration (HPSD) ablation following the CLOSE protocol represents a common ablation modality for PVI so far.^3–5^ In the recent years, RF ablation has been constantly optimized, improving clinical outcome.^6,7^ However, achieving effective and safe PVI might be challenging, as recurrences are still common and RF ablation can cause severe collateral damage, such as atrio-oesophageal fistula formation or phrenic nerve palsy.^8,9^ Since 2021, pulsed field ablation (PFA) is commercially available in Europe as a potential alternative to RF ablation in patients with AF. High-voltage, microsecond impulses can create transmural lesions by destabilizing the cell membrane and forming irreversible nanoscale pores and leakage of cell contents, which will ultimately lead to cell death.^10^ Due to its relative tissue selectivity, it is possible to selectively target myocardial tissue by means of PFA whilst sparing the surrounding non-cardiac tissue. Since 2018, several clinical studies have reported on efficacious and safe ablation using PFA for PVI in patients with paroxysmal AF.^11–14^ The first long-term data report an efficacy of arrhythmia-free survival after 1 year ranging from 70 to 85%.^14,15^ Recently, the first randomized trial comparing conventional thermal ablation with PFA was published.^16^ However, real-world data outside randomized trials comparing the long-term effectiveness of PFA-PVI with HPSD-RF PVI are still scarce. Here, we report the largest single-centre, real-world experience comparing the acute efficiency, safety, and long-term outcome of both techniques in a cohort of patients with paroxysmal AF undergoing PVI.

## Methods

We performed a retrospective, non-randomized single-centre study based on our institutional electronical registry database, including consecutive all-comer patients with symptomatic, paroxysmal AF undergoing PVI with a standardized HPSD-RF or PFA protocol at the Alfried Krupp Hospital in Essen, Germany, between June 2019 and December 2022 (Pulsed Field veRsus hIgh-power short-duratiOn Radiofrequency ablatIon, PRIORI study). All procedures before April 2021 were only performed using HPSD-RF ablation. As the pentaspline PFA catheter gained CE approval in March 2021, our centre started using this system from April 2021 onwards. Since this study intended to compare the success of PVI only, patients who underwent ablation beyond PVI were excluded. Pathophysiologically, the PVs have been described as the main site harbouring triggers initiating AF paroxysms. Therefore, PVI is the primary treatment strategy in patients undergoing percutaneous AF ablation, particularly among paroxysmal AF cases where the likelihood of the PVs being the culprit sources of ectopic beats initiating AF is significantly higher that non-paroxysmal AF patients. With regard to efficacy and safety, we wanted to have a comparable population. Results might highly differ when including both paroxysmal and persistent AF. This might have falsified the results. Each patient provided written informed consent or each interventional procedure and scientific data collection. The principle outlined in the latest update of the Declaration of Helsinki was followed. Paroxysmal AF was defined as episodes lasting <7 days or episodes that were cardioverted within 7 days.^17^

### Procedure

On the day of the ablation procedure, patients remained in a fasting state. Pulmonary vein isolation was performed under deep conscious sedation utilizing midazolam, oxycodone, and a continuous infusion of propofol. Vitamin K antagonist treatment was uninterrupted aiming for an international normalized ratio between 2 and 3, whilst non-vitamin K antagonists were omitted on the morning of the procedure. At the end of the procedure, all catheters were removed. Transthoracic echocardiography was performed to rule out pericardial effusion. The right femoral puncture site was closed with a Z-suture (until the next morning), and a pressure bandage was applied for 6 h. Using haemodynamic monitoring, the patient was allowed to recover.

### High-power short-duration radiofrequency ablation protocol

All patients underwent transoesophageal echocardiography prior to the procedure to exclude left atrial (LA) thrombus. After triple right femoral vein puncture, a decapolar electrode catheter (Webster CS, Biosense Webster, Inc., Irvine, CA, USA) was inserted in the coronary sinus. Under fluoroscopic guidance, a double transseptal puncture was performed using 8.5 French (F) sheaths (SL1 Fast-Cath Guiding Introducer, St. Jude Medical, Minneapolis, MN, USA). Directly after the transseptal puncture, intravenous unfractionated heparin boluses were administered to maintain an activated clotting time of >350 s. The sheaths were continuously irrigated with heparinized saline. After transseptal access, a 3D electroanatomic map (EAM) of the left atrium and PVs was constructed using a non-fluoroscopic navigation system (Carto 3®, Biosense Webster, Inc., Diamond Bar, CA, USA) and a pentaspline mapping catheter (Pentaray, Biosense Webster, Inc., Diamond Bar, CA, USA). If the patient was in AF, electric cardioversion was performed to restore sinus rhythm before mapping. Fast anatomic mapping (FAM) was performed in all patients. RF applications were delivered using a 3.5 mm Thermocool Smarttouch SF® catheter (Biosense Webster, Inc., Diamond Bar, CA, USA) in power control mode. Radiofrequency power was set to 45 W, and the catheter tip was irrigated by saline at a flow rate of 2 mL/min during mapping and 15 mL/min during ablation, respectively. Application of 45 W was considered as HPSD according to previous publications, but not as very HPSD.^18^ Ablation was performed by adhering to the CLOSE protocol.^4^ Radiofrequency energy was delivered until an ablation index (AI) of 450 at the posterior wall/inferior/roof and 600 at the anterior wall was reached. The continuity of both ablation circles was further checked using complete transparency of the voltage map. Upon completion of circumferential ablation, the pentaspline mapping catheter was used to demonstrate both entrance and exit blocks to confirm bidirectional PV isolation. In the case of absence of isolation after completion of the circle, touch-up ablation was delivered until bidirectional PV isolation was achieved.

### Pulsed field ablation protocol

All patients underwent cardiac CT angiography prior to the procedure to exclude LA thrombus, to measure all PV ostial diameters, and to obtain a detailed understanding of the LA anatomy. After double right femoral vein puncture, a decapolar electrode catheter (Webster CS, Biosense Webster, Inc., Irvine, CA, USA) was inserted in the coronary sinus. Under fluoroscopic guidance, a single transseptal puncture was performed using an 8F sheath (SR0 Fast-Cath Guiding Introducer, St. Jude Medical, Minneapolis, MN, USA). Directly after the transseptal puncture, intravenous unfractionated heparin boluses were administered to maintain an activated clotting time of >350 s. After transseptal puncture, the 8F sheath was exchanged for a 13F inner diameter, 16.8F outer diameter transparent sheath (Faradrive, Farapulse, Menlo Park, CA, USA). The sheath was continuously irrigated with heparinized saline. Initially, a 0.035 inch, 180 cm extra-stiff, straight-tip guidewire (Amplatz Extra Stiff, Cook Medical, Bloomington, IN, USA) was used as a rail to deploy the multielectrode 12F over-the-wire PFA catheter (Farawave, Farapulse, Menlo Park, CA, USA) into the desired shape and advanced it into position at the antrum of each PV. During the study period, the straight guidewire was replaced by a J-tip guidewire (InQwire, Merit Medical System, Inc., South Jordan, UT, USA). Two catheter sizes were available: 31 and 35 mm in maximal diameter at full deployment. The choice of the catheter size was dependent on the maximal PV diameter in the LA CT angiography. In case of a PV > 27 mm diameter from superior to inferior, the 35 mm PFA was chosen. Ablative energy was delivered from all electrodes, recording electrograms, and pacing was possible through the third electrode of each spline. Ablation procedural workflow was described in detail previously.^11,12^ In brief, eight biphasic pulse trains with a power of 1900 or 2000 V were applied per PV, that is, four applications each in the basket/biscuit and flower poses. Between the first pair of applications and the second pair of applications in a particular pose, the over-the-wire catheter was rotated 30–40°. Using the Carto SEG (Biosense Webster, Inc.) software module, the 3D LA and PV reconstruction of the CT angiography was used as a roadmap to fluoroscopically position the guidewire and the PFA catheter. Selective PV angiographies and biplane fluoroscopy were used to optimize contact between the PFA catheter and tissue and ensure adequate coverage of the PV antra for all patients. Additional pulsed field applications were performed at the operator’s discretion. No additional ablations outside of the PV were applied. Acute isolation of the treated PV was determined by the mapping electrode on each spline of the PFA catheter. A PV stimulation to test for exit block was not performed.

### Clinical follow-up and study outcome

Patient characteristics and procedural data were documented in the database and during follow-up. The patients were scheduled for clinical and 5-day Holter ECG follow-up at 3, 6, and 12 months. Any anti-arrhythmic medication with exception of ß-blocker was stopped 3 months after the initial ablation procedure if acceptable. Oral anti-coagulation was continued for 3 months in all patients and afterwards according to stroke risk using the CHA_2_DS_2_-VASc score. Ablation was deemed successful in the absence of symptomatic or asymptomatic atrial tachyarrhythmias lasting more than 30 s identified on surface ECG or on Holter monitoring, off anti-arrhythmic drug therapy. As early relapse of atrial tachyarrhythmias within the first 90 days after ablation may be a transient phenomenon, this transition period was excluded from the final analysis.^19^

### Statistical analysis

Continuous variables are presented as mean and SD or median and interquartile range (IQR; 25th–75th percentiles) where appropriate. The Student’s *t*-test or the Mann–Whitney *U* test was used for unpaired group comparison of continuous data. Categorical variables were compared by *χ*^2^ or Fisher’s exact test and were presented as frequency and percentage. The Kaplan–Meier estimate was used to compare freedom from atrial arrhythmias after ablation. All tests were two sided, and a *P* < 0.05 was considered statistically significant.

## Results

### Patient characteristics

A total of 410 patients (201 PFA, 209 HPSD-RF) who received first time PVI for paroxysmal AF at our institution were included. The baseline characteristics were comparable between both groups. Most patients were of male sex (PFA, 56%; HPSD-RF, 53%), the median age was 68 (59–73) years in PFA and 68 (59–75) years in HPSD-RF (*P* = 0.722). Further baseline characteristics did not differ between the two groups and are summarized in *Table 1*.

**Table 1:** Patient characteristics

	PFA, *n* = 201	HPSD-RF, *n* = 209	*P*-value
Age (years)	68 [59–73]	68 [59–75]	0.722
Sex (male, %)	56 (114)	53 (111)	0.448
BMI (kg/m^2^)	27 [25–30]	27 [25–31]	0.272
CHA_2_DS_2_-VASc score	2 [1–4]	2 [1–3]	0.645
Hypertension, *n* (%)	133 (66)	141 (67)	0.834
Diabetes mellitus, *n* (%)	22 (11)	27 (13)	0.547
Previous stroke or TIA, *n* (%)	11 (5)	22 (11)	0.07
Coronary artery disease, *n* (%)	19 (9)	21 (10)	0.869
AAD, *n* (%)	46 (23)	43 (21)	0.718

Values are presented as *n* (%) or median [IQR]. CHA_2_DS_2_-VASc score: congestive heart failure, hypertension, age ≥75 (2 points), diabetes mellitus, stroke (2 points), vascular disease, age 65–75, and sex category.

AAD, anti-arrhythmic drugs; BMI, body mass index; HPSD, high-power short-duration; PFA, pulsed field ablation; RF, radiofrequency; TIA, transient ischaemic attack.

### Procedural characteristics

Complete PVI was successful in all 410 patients. In both groups, all patients were in sinus rhythm after ablation. A 35 mm PFA catheter was used in 16/201 (8%) of the patients. Procedural data are displayed in *Table 2*. Median procedure times were significantly shorter in the PFA group compared to the HPSD-RF group [61 (44–103) vs. 125 (105–143) min, *P* < 0.001]. The LA dwell time was significantly shorter in the PFA group compared to the HPSD-RF group [46 (34–92) vs. 110 (92–125) min, *P* < 0.001]. Fluoroscopy time was significantly longer in the PFA group compared to the HPSD group [16 (13–20) vs. 4 (2–5) min, *P* < 0.01]. Correspondingly, the overall fluoroscopy dosage was significantly higher in the PFA group compared to the HPSD-RF group [412 (270–739) vs. 129 (58–265) μGym^2^, *P* < 0.001].

**Table 2:** Procedural data

	PFA, *n* = 201	HPSD-RF, *n* = 209	*P*-value
Procedural time, min	61 [44–103]	125 [105–143]	<0.001
LA dwell time, min	46 [34–92]	110 [92–125]	<0.001
Fluoroscopy time, min	16 [13–20]	4 [2–5]	<0.001
Fluoroscopy dose, μGym^2^	412 [270–739]	129 [58–265]	<0.001

Values are presented as median [IQR].

HPSD, high-power short-duration; LA, left atrium; min, minutes; PFA, pulsed field ablation; RF, radiofrequency.

### Safety

Safety data are displayed in *Table 3*. The overall complication rates were 6/201 (2.9%) in the PFA group and 13/209 (6.2%) in the HPSD-RF group and did not differ significantly (*P* = 0.159). There was one stroke and one minor TIA in the PFA group (1.0%). The stroke subsequently led to death (81-year-old female, CHA_2_DS_2_-VASc score of 5). In the PFA group, three (1.4%) tamponades were recorded, mainly during the initial phase using the extra-stiff, straight-tip guidewire. All tamponades were managed by pericardiocentesis. After switching to the J-tip guidewire, no further tamponades were recorded. One minor access site complication (1%) without any clinical consequence was reported in the PFA group.

**Table 3:** Procedural complications

	PFA, *n* = 201	HPSD-RF, *n* = 209	*P*-value
Access site, *n* (%)	1 (0.5)	3 (1.4)	0.623
Tamponade, *n* (%)	3 (1.4)	6 (2.8)	0.504
Stroke or TIA, *n* (%)	2 (1.0)	2 (0.9)	1.000
Air embolism, *n* (%)	0	2 (0.9)	0.499
Total, *n* (%)	6 (2.9)	13 (6.2)	0.1587

Values are presented as *n* (%).

HPSD, high-power short-duration; PFA, pulsed field ablation; RF, radiofrequency; TIA, transient ischaemic attack.

There were two strokes (0.9%) in the HPSD-RF group. There were six (2.8%) tamponades requiring pericardiocentesis. In two patients (0.9%), transient ST-segment elevation without any clinical consequence was reported. Access site complications were reported in three (1.4%) patients of the HPSD group. It should be noted that no ultrasound-guided puncture was performed in either the PFA group or the HPSD-RF group. No PFA- or RF-specific complications such as atrio-oesophageal fistula, phrenic nerve palsy, or PV stenosis were identified.

### Procedural outcome and redo procedures

**Figure 1:**
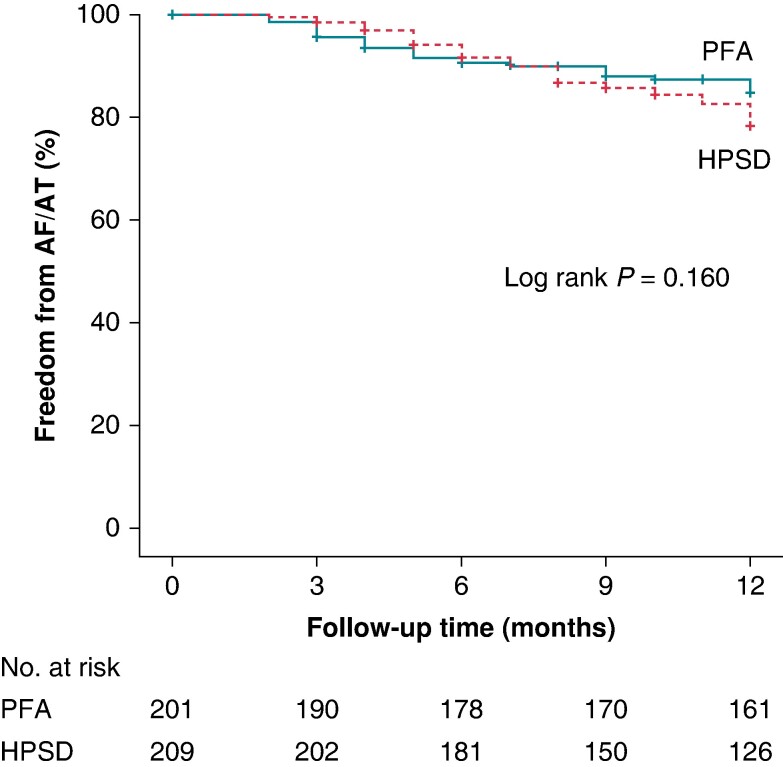
Kaplan–Meier analysis. The graph shows Kaplan–Meier estimates of freedom from any atrial tachyarrhythmia episodes lasting >30 s after a single procedure. AF, atrial flutter; AT, atrial tachyarrhythmia; HPSD, high-power short-duration; PFA, pulsed field ablation.

There was no significant difference for freedom from any atrial arrhythmia after a single procedure between groups. In total, 85% patients of the PFA group were free from arrhythmia recurrences vs. 79% patients in the HPSD-RF group. For a subgroup of patients (*n* = 41, 20%) in the HPSD-RF group, only transtelephonic follow-up was possible due to the COVID-19 pandemic and limited outpatient clinic options. After a median follow-up of 365 days (IQR: 331–383), the Kaplan–Meier analysis (*Figure 1*) showed no significant differences in arrhythmia-free survival between both groups (log-rank *P* = 0.16).

Overall, there were 30 (15%) recurrences in the PFA group and 44 (21%) recurrences in the HPSD-RF group. In the PFA group, 22 patients (73%) underwent a redo procedure, which showed all veins permanently isolated in 6/22 (27%) of the patients and 54/77 (70%) of the veins durably isolated. In the HPSD-RF group, a redo procedure was performed in 37/44 (84%) patients. In this group, all veins were isolated in 9/37 (24%) patients, and 103/166 (62%) of the veins were permanently isolated. The most common site of reconnection was the left superior PV for PFA (33%) and for HPSD-RF (43%; *P* = 0.566). No differences with respect to PV reconnection of the left inferior PV (PFA, 17%; HPSD-RF, 32%; *P* = 0.335), right superior PV (PFA, 27%; HPSD-RF, 21%; *P* = 0.701), or right inferior PV (PFA, 24%; HPSD-RF, 27%; *P* = 0.953) were seen.

## Discussion

In this single-centre study, we aimed to evaluate the procedural characteristics, safety, and 1-year outcome in 410 patients who underwent PVI for paroxysmal AF using either PFA or HPSD-RF ablation. The major findings are as follows:

1. Pulsed field ablation and HPSD-RF are very effective for acute PVI with 100% success in both groups.
2. Pulsed field ablation showed significant shorter procedure times; however, fluoroscopy time and dosage were higher.
3. No significant difference in safety profile in both groups.
4. Atrial arrhythmia-free survival was similar after 1-year follow-up (PFA, 85%; HPSD-RF, 79%).
5. At the time of repeat ablation, PFA patients showed a non-significant different number of reconnected PVs compared to those initially treated with HPSD-RF.
6. The most common site of reconnection was the left superior PV.

### Procedural efficacy

We observed a very high overall procedural efficacy both for PFA and HPSD-RF ablation. This is similar to what has been reported previously using these different modalities for PVI in paroxysmal AF.^3,12–14^ From a technical perspective, procedural times were significantly lower using the PFA system compared to the HPSD-RF group. The overall short procedural times highlight the advantages of PFA as a ‘single-shot’ device in daily routine and are in line with various other publications.^13,14,20–22^ With the availability of EAM-supported PFA systems on the market, the current time advantage of fluoroscopy-only-guided systems must certainly be re-evaluated.^23,24^ Despite shorter procedure times, fluoroscopy time and dosage were significantly higher in the PFA group. The limited use of fluoroscopy in the HPSD-RF group may be due to the extensive experience of the operators involved, but it also shows that there is still room for improvement when using PFA. However, fluoroscopy time should decrease as familiarity with PFA increases. It has been shown that operator experience reduces fluoroscopic exposure during PFA.^25^ In addition, the fluoroscopy times are likely to be further reduced using EAM. First, EAM-based PFA systems demonstrated favourable fluoroscopy times.^23,24^ However, as radiation exposure during catheter ablation procedures may have a long-term adverse impact on both the patients and the medical staff, the fluoroscopy times and doses that occur during RF ablation are still the benchmark.

### Procedural outcome

To our knowledge, this study is one of the largest to compare PFA and HPSD-RF in paroxysmal AF and highlights the acute and long-term efficacy of both techniques following a standardized institutional ablation procedure protocol. Our data are in line with what has been previously reported. Two large registries, the MANIFEST-PF and the EU-PORIA multicentre registries, demonstrated an arrhythmia-free survival of 81 and 80% in paroxysmal AF using PFA, respectively.^13,14^ The reported high efficacy rates for RF ablation align with prior CLOSE studies that used thermal energy for contiguous PV encirclement.^4,26^ In the recently published ADVENT trial, the 1-year arrhythmia-free survival for paroxysmal AF was lower for PFA (73%) and RF ablation (71%).^16^ This lower efficacy rate may be explained due to the large number of centres and different operators, most of which had little or no experience with PFA. However, the EU-PORIA registry showed that freedom from any arrhythmia was not influenced by operator experience and that there is a steep learning curve among operators with limited experience in PFA.^13^ In the recently published FARA-Freedom study by Metzner *et al.* with a more stringent rhythm monitoring and a rigorous definition of treatment success, the 1-year efficacy was 66.6%. Recurrences can be caused by multiple factors, but PV reconnection remains a major cause. Despite the comparable clinical outcome in both groups, we observed a statistically non-significant trend towards a higher rate of permanently isolated PVs in favour of PFA. The PV reconnection rate was 30% after PFA and 38% after HPSD-RF in our study. In a recently published study by Della Rocca *et al.*,^27^ they showed a significantly lower number of reconnected PVs in PFA (19.1%) compared to HPSD-RF ablation (34.8%). The authors showed the left superior PV to be the most frequently reconnected PV after PFA in 27%. These data are in line with our data. We showed that the LSPV was reconnected in 33%. Similar findings were also reported from previously published studies focusing on high-density mapping.^27–29^ In a recently published study by Kueffer *et al.*^30^ in 144 patients, durable isolation was observed in 71% of the PVs during a redo procedure, and 38% of all patients showed a durable isolation of all veins. In a recent publication, interestingly, there were significantly more repeat ablations in patients with a left common pulmonary vein (LCPV), although 60% remained durably isolated. As the number of LCPV and redo procedures was low in our cohort, it is not possible to meaningfully assess the significance of an LCPV within our work. Further studies are required to investigate the types of reconnections after PFA. Whether different catheter designs or optimization in waveforms may improve lesion depths and durability needs to be further investigated.

### Safety

Our study highlights the favourable safety profile of both PFA and HPSD-RF ablation. The complication rate was low and comparable to previous data. There were three cardiac tamponades in the PFA group. All tamponades occurred whilst using the extra-stiff, straight-tip guidewire. This possible cause has been extensively discussed before.^20,22,31^ Therefore, practice changed resulting in abandoning the use of this certain guidewire and the use of a J-tip guidewire instead. After switching to the J-tip guidewire, no further tamponades occurred in the PFA group. In addition, in the observational MANIFEST-PF study, the incidence of pericardial tamponade seemed to decrease with increasing operator experience.^32^ Other serious complications included three strokes in the overall population of 410 patients (0.73%) and one TIA in the PFA group (0.49%). Both cerebral complications in the PFA group occurred in the early phase after implementation of the catheter, which might be explained by sheath management and air embolism. However, we have to await the results of more randomized controlled trials (such as BEAT-AF) also comparing the safety of PFA with that of RF in larger patient cohorts, with specific emphasis on silent adverse events such as silent brain injury we did not account for in this study.^33^ The vascular complication rate was low in both groups. However, ultrasound-guided puncture was not performed during the study period in both groups and might reduce complications further. Importantly, despite the use of a 16.8F outer diameter sheath in the PFA group, the access site complication rate was low. However, since HPSD-RF ablation requires three access site punctures and double transseptal punctures, the numerically greater number of complications might be a result of the procedure technique.

### Limitations

Our findings must be interpreted in the light of several limitations. First, this was a high-volume single-centre, retrospective study. Second, our study is limited by its non-randomized design and sample size. A bias in patient selection, therefore, cannot be excluded. Third, all patients in our study had paroxysmal AF. Our findings therefore cannot be extended to patients with non-paroxysmal AF. Fourth, we did not use implantable loop recorders due to the retrospective design of the study. Follow-ups were mainly based on 5-day Holter ECG recordings representing clinical standard in all comparative studies including thermal energy and PFA. As no implantable loop recorders were used, some episodes of asymptomatic recurrence of AF may have been missed. However, in contrast to the results of other recent trials, our results represent comparable single-procedure outcomes. During the study, the power per pulse train was increased from 1900 to 2000 V. This might have affected the outcome in the PFA group. Finally, we did not check for the exit block in the PFA group. However, recent publications demonstrated non-inferiority when not checking for the exit block in PFA procedures.

## Conclusions

Pulsed field ablation and HPSD-RF were highly effective and safe in achieving PVI in the setting of paroxysmal AF. The arrhythmia-free survival is comparable. Pulsed field ablation procedures were shorter, which might be of additive value in a clinical setting. However, fluoroscopy time and dosage were higher. Overall complication rates were similar.

## Data Availability

The data underlying this article will be shared on reasonable request to the corresponding author.
